# Insights about the common generative rule underlying an information foraging task can be facilitated via collective search

**DOI:** 10.1038/s41598-022-12126-3

**Published:** 2022-05-16

**Authors:** Aoi Naito, Kentaro Katahira, Tatsuya Kameda

**Affiliations:** 1 Department of Social Psychology, The University of Tokyo, Tokyo, 113-0033 Japan; 2 Japan Society for the Promotion of Science, Tokyo, 102-0083 Japan; 3 Human Informatics and Interaction Research Institute, National Institute of Advanced Industrial Science and Technology (AIST), Tsukuba, 305-8566 Japan; 4 Brain Science Institute, Tamagawa University, Tokyo, 194-8610 Japan; 5 Center for Experimental Research in Social Sciences, Hokkaido University, Sapporo, 060-0810 Japan

**Keywords:** Psychology, Human behaviour

## Abstract

Social learning is beneficial for efficient information search in unfamiliar environments (“within-task” learning). In the real world, however, possible search spaces are often so large that decision makers are incapable of covering all options, even if they pool their information collectively. One strategy to handle such overload is developing generalizable knowledge that extends to multiple related environments (“across-task” learning). However, it is unknown whether and how social information may facilitate such across-task learning. Here, we investigated participants’ social learning processes across multiple laboratory foraging sessions in spatially correlated reward landscapes that were generated according to a common rule. The results showed that paired participants were able to improve efficiency in information search across sessions more than solo participants. Computational analysis of participants’ choice-behaviors revealed that such improvement across sessions was related to better understanding of the common generative rule. Rule understanding was correlated within a pair, suggesting that social interaction is a key to the improvement of across-task learning.

## Introduction

Learning how to adjust decision strategies under uncertainty through repeated experiences constitutes a fundamental problem both in modern human societies (e.g., consumer choices, financial decisions, or scientific enquiries) and in natural environments (e.g., searching for food, water, and shelter). In particular, given limited time and energy budgets, individual decision makers often must strike a balance between acquiring new information (“exploration”) and harvesting to the best of their current knowledge (“exploitation”) to maximize overall expected outcomes from their choices. The ubiquity of this exploration–exploitation tradeoff has prompted a wide range of research from animal foraging to computer science^1–3^. Previous research has also shown that decision makers interacting with others can increase their decision performances collectively, through decentralized information pooling affected by various social learning strategies^4–9^.

**Figure 1:**
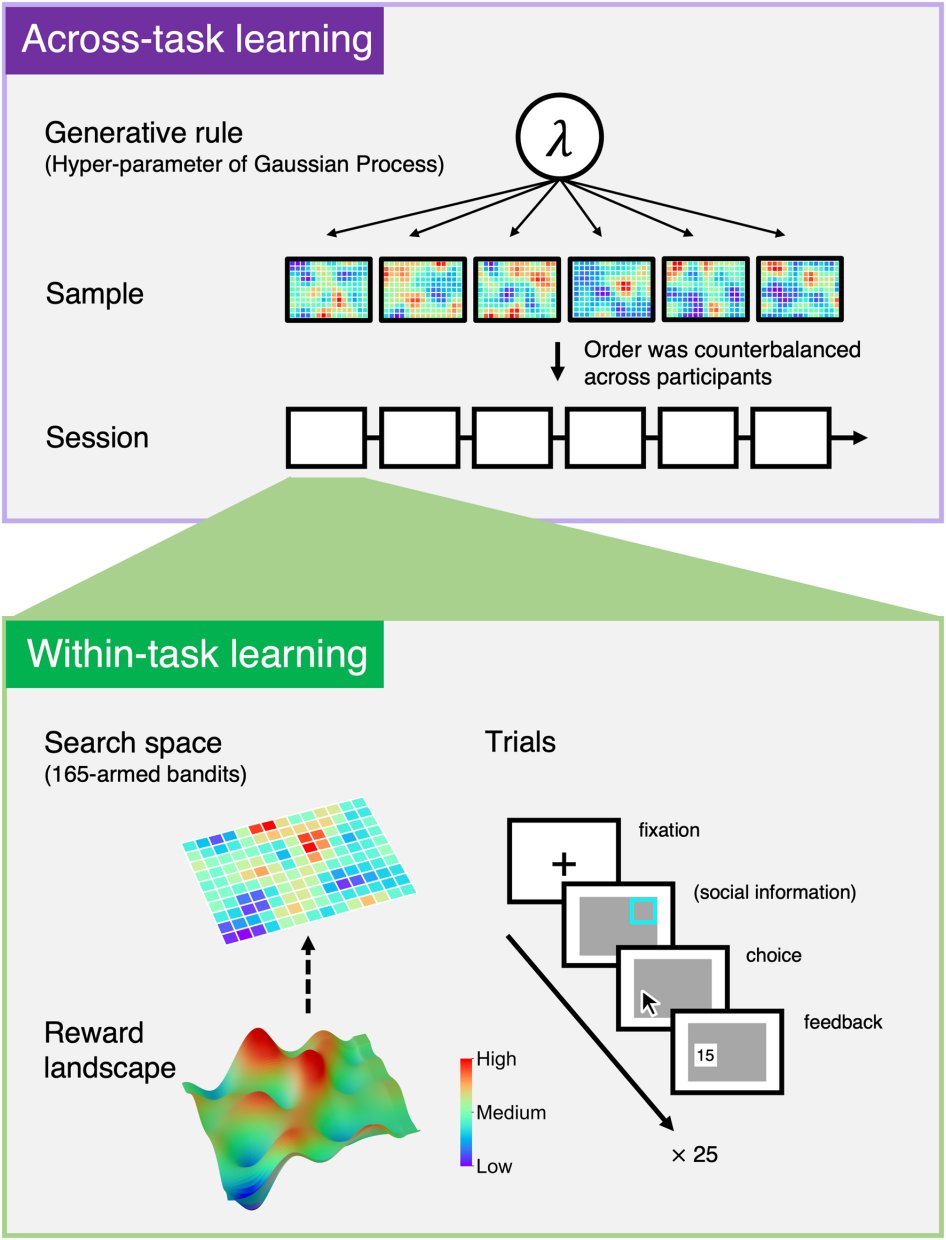
Across- and within-task learning in the spatially correlated multi-armed bandit (MAB). (Top) Across-task learning: Prior to the experiment, six environments (reward landscapes) were independently sampled from a gaussian process prior with radial basis function kernel with a single length-scale parameter $\lambda$ (= 1.5 in the experiment; Supplementary Fig. S12). For each of the six environments, we rescaled reward values to range from 0 to a maximum value that was randomly generated from a discrete uniform distribution [80, 120]. In other words, the value of the global optimum was different across environments so that participants could not infer it from experience in a previous environment. In the experiment, the six environments were assigned to six sessions randomly across participants, with the constraint that those in the same pair experienced the same sequence of the environments. Thus, participants in both the solo and pair conditions experienced the six variations of the reward landscape in a randomized order across six sessions. If participants developed a general knowledge about the common structure gradually across the sessions (across-task learning), then they could solve the specific exploration–exploitation tradeoff in each environment more efficiently in later sessions. (Bottom) Within-task learning: In each of the six sessions, all participants worked on the 165-armed bandit task (displayed as a 11 × 15 grid on a computer screen) for a total of 25 trials. Each box in the 11 × 15 grid corresponds to an option whose color represents the magnitude of mean rewards (e.g., red boxes indicate options with high rewards while violet boxes indicate those with low rewards, according to the rainbow colormap). Notice that the number of options (= 165) was much larger than the total number of trials within a session (= 25), during which they had to learn to find good (if not necessarily the best) options in the current environment (within-task learning).

In the real world, however, the possible search space is often so huge that decision makers are incapable of encountering or evaluating all options, even if they can pool information collectively. One potential strategy to cope with the overflow is developing some cognitive model about the environment that systematically guides information search under uncertainty. This involves cognitively inferring the proximate structure of the current environment (“reward landscape”) from available/sampled data (“within-task” learning; Fig. 1 bottom). Additionally, if similar decisions are repeated in a series of environments, decision makers may also examine whether and how knowledge about the old environment may be generalizable to new environments by inferring some common generative rule (“across-task” learning; Fig. 1 top). If such generalization is warranted (i.e., the old and new environments are structured or generated according to a common rule^10–13^), decision makers can solve the exploration–exploitation tradeoff in the new environments more efficiently. Although the question of knowledge generalizability has been discussed for many years^14,15^, it has remained largely unanswered because of the computational difficulty of quantifying its cognitive underpinnings in detail. However, recently developed techniques in machine learning^16^ have enabled computational approaches to assess cognitive mechanisms for knowledge generalization at a finer level^17–20^.

On the other hand, to the best of our knowledge, these new developments are concerned only with individual decision making, and insights about how people may incorporate social information into across-task learning through decentralized information pooling remain elusive. For example, most previous research on social learning has focused on imitation learning, investigating when and how learners copy others’ preceding choices in the current environment^8,21–28^. Certainly, such imitation learning allows learners to efficiently acquire locally useful knowledge about the reward landscape in the current environment (within-task learning). However, even if local knowledge obtained through imitation learning is highly accurate, it remains unknown how such local knowledge may be extendable to a new environment. To empirically assess how the use of social information may possibly enhance not only within-task learning but also across-task learning^10,11^, we need to create a new testbed where a group of individuals simultaneously participate in a series of task environments that follow a common generative rule but have random components specific to each environment.

In this study, we investigated social learning processes in a large search space, using multi-armed bandit (MAB) tasks with 165 options. As illustrated in Fig. 1 bottom, the 165 options in the search space were arrayed in a 11 × 15 grid on a computer screen, where rewards from more nearby options were more similar. Following previous studies about individual decision making^18–20^, we generated such a spatially-correlated reward landscape using a Gaussian process, which is a widely-used variant of machine learning models (Fig. 1 top). If participants developed some understanding of the spatially-correlated structure of the environment, it would allow them to do more systematic exploration under uncertainty, rather than simple random exploration^29^. Participants worked on a total of six experimental sessions (Fig. 1 top), each composed of 25 trials (Fig. 1 bottom), in which the total number of search opportunities (= 25) was much fewer than the number of options (= 165). Prior to the experiment, we created each search space following a common generative rule to implement in all six sessions (i.e., we sampled each space independently from the Gaussian process prior with $\lambda$ = 1.5; Fig. 1 top).

In the experiment, we had solo and pair conditions. Upon arrival at the laboratory, each participant was seated in a private soundproof cubicle and received further instructions on a computer monitor. No direct communication between participants was allowed at any point during the experiment. Before the main sessions, all participants worked on the same practice task (i.e., the 165-armed bandit task; Supplementary Fig. S1) individually for 10 trials with feedback. In the main sessions, forty-seven participants worked on the task alone, while 74 participants worked in pairs (i.e., 37 pairs in total). Prior to making a choice in each trial, participants in the pair condition were provided information about their partner’s choice in the preceding trial in the form of a visual cue highlighting the chosen option (but not the partner’s reward information, which was available only to the partner). After making a choice, participants in both conditions received individual feedback about their reward in the trial (Fig. 1 bottom).

In the following, we examine how and to what extent social learning may facilitate not only within-task learning about a particular environment, but also across-task learning about multiple related environments. We are concerned with whether and how the social-learning opportunities in the pair condition may help participants to develop general knowledge about the common structure of the environments, as well as to solve the exploration–exploitation tradeoff specific to each environment more efficiently. To address this question, we first compare trajectories of performance across the six main sessions between the solo and pair conditions. We then analyze each participant’s choice behaviors using computational models. The computational modelling allows us a finer analysis of the possibility of across-task learning by dissociating the key parameter that corresponds to understanding of the common generative rule from other parameters that correspond to imitation learning and basic choice strategies for solving the exploitation-exploration tradeoff under uncertainty.

## Results

### Behavioral performance

**Figure 2:**
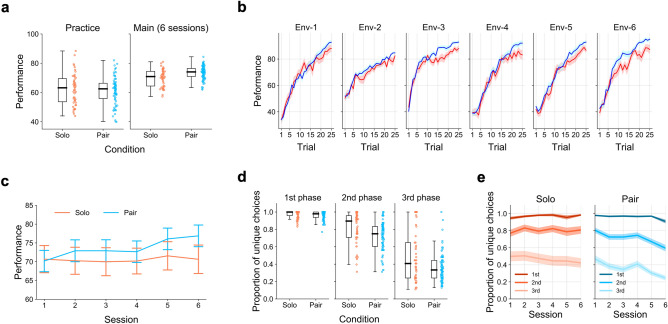
Behavioral performance. (**a**) Performance in the practice session (left) and performance collapsed across the main 6 sessions (right). Each dot represents one participant. (**b**) Participants’ mean performance over 25 trials in each of the six environments. The colored traces represent mean performance in the two conditions (red: solo, blue: pair), and the shaded areas show the standard errors of the mean. Each panel corresponds to one of the six environments shown in Supplementary Fig. S11; their order was counterbalanced across participants. (**c**) Trajectories of mean performance over the 6 sessions. Error bars show standard errors of the mean. (**d**) Proportions of unique choices in the first (trial 1–8), second (trial 9–16), and third (trial 17–25) phases. Each dot represents one participant’s mean in each phase. (**e**) Trajectories of the proportion of unique choices in each phase across sessions for the solo and pair conditions. The dark, intermediate, and light colored lines stand for means in the first (trial 1–8), second (trial 9–16), and third (trial 17–25) phases, respectively. The shaded areas show the standard errors of the mean.

In Fig. 2a, we show participants’ performance in terms of average points earned during the six sessions. Collapsed across all sessions, participants in the pair condition performed better than those in the solo condition (Linear Mixed Model (LMM), condition: 95% CI [1.41, 5.16], mean = 3.28; Fig. 2a right). There was no performance difference in the practice session, in which participants worked on the task individually for 10 trials with feedback (LMM, condition: 95% CI [− 5.14, 2.56], mean = − 1.34; Fig. 2a left). These patterns are in line with the performance improvement due to social learning in simpler MAB environments without spatial correlations^7^. As shown in Fig. 2b, the difference in performance between the solo and pair conditions across all six environments increased in later trials within each session (LMM, condition × trial: 95% CI: [0.04, 0.47], mean = 0.26). This indicates that, compared to individual learning (i.e., the solo condition), social learning opportunities helped participants find and exploit better options relatively earlier, consequently achieving greater overall performance in the current environment. Interestingly, the proportion of sessions (out of 6) in which each participant found the optimal option over 25 trials was not different between the two conditions (Generalized Linear Mixed Model (GLM), condition: 95% CI [− 0.06, 0.53], mean = 0.23; Supplementary Fig. S2), suggesting that social learning helped participants cope with the exploration–exploitation tradeoff by settling on better options while avoiding the risk of a wasteful search for the global optimum.

Most importantly, the advantage of social learning (the pair condition) over individual learning (the solo condition) was manifested more clearly in later sessions in the task sequence (Fig. 2c). The interaction effect between session progress and condition was significant (LMM, session × condition: 95% CI [0.31, 2.06], mean = 1.21; see also Supplementary Fig. S3). As seen in Fig. 2c, participants in the pair condition became more adept at solving the exploration–exploitation efficiently in later sessions, while this was not the case in the solo condition. These patterns are in line with our conjecture that social learning opportunities may promote not only within-task learning but also across-task learning. Later, we revisit these notions more directly by introducing several computational models to analyze each participant’s choice behavior.

### Exploration pattern

To obtain further insights about how social learning opportunities are used in search decisions under the exploration–exploitation tradeoff, we also examined participants’ exploration patterns. As a behavioral metric of exploration, we examined proportions of unique (i.e., once-only, “new”) choices that each participant made in each session. Because the desirable balance between exploration and exploitation should depend on the progress of trials, we divided each session into three phases (the first phase from trial 1 to 8, the second from trial 9 to 16, and the third from trial 17 to 25), and counted the number of unique choices by each participant. Collapsed across the six sessions, the average proportion of unique choices gradually decreased from the first to the third phases (Fig. 2d). The proportion of unique options was no different between the two conditions in the first phase (GLMM, condition: 95% CI [− 1.32, 0.26], mean: − 0.52), whereas it was lower in the pair condition than in the solo condition for the second (GLMM, condition: 95% CI [− 1.34, − 0.34], mean = − 0.84) and third phases (GLMM, condition: 95% CI [− 1.18, − 0.22], mean = − 0.7). This means that the participants in the pair condition explored the environment more efficiently in earlier trials, engaging in more exploitation in later trials (see also Supplementary Fig. S4 for related results about the mean migration length from one trial to the next as another measure of participants’ exploration behaviors).

We further examined how this pattern may have evolved across the six sessions. In Fig. 2e, we decomposed Fig. 2d to show trajectories of proportions of unique choices across sessions, for the first, second, and third phases respectively. We examined whether participants exhibited different trajectories across sessions between the solo and pair conditions by focusing on the condition × session interaction. The GLMM revealed significant condition × session interactions for the first and second phases (GLMM: 95% CI for the first phase [− 0.97, − 0.25], mean = − 0.6; second phase [− 0.45, − 0.07], mean = − 0.26), but not for the third phase ([− 0.24, 0.04], mean = − 0.1). That is, as the sessions progressed, participants in the pair condition switched to exploitation in earlier phases compared to those in the solo condition. These results again suggest greater across-task learning of the common environmental structure in the pair condition than in the solo condition (see also Supplementary Fig. S5 for exploratory analysis of participants’ subjective understanding of the environmental structure).

### Computational accounts of behavior

The results so far are in line with our conjecture that social learning opportunities may facilitate not only within-task learning but also across-task learning. To examine these results at a finer level, we introduce a computational model of participant decision processes. As we have discussed earlier, copying the partner’s choice in the current environment^21,22^ does not necessarily explain how enriched local knowledge (within-task learning) can be extended to a new environment (across-task learning).

Here, we consider a decision model with Gaussian Process regression and an Upper Confidence Bound policy (GP-UCB model hereafter). The GP-UCB model has been empirically verified in several studies about human exploratory behavior in spatially-correlated environments as in Fig. 1^18–20^. In the following, we outline each component of the model and discuss the interpretation of each parameter (for details of the model, see Methods and Supplementary Information).

The GP-UCB model has two elements: GP, which represents the spatial structure of the environment, and UCB, which represents a person’s choice policy in the environment.

The first element, Gaussian Process (GP) regression, generates the spatial distribution of rewards in the environment, which is characterized by the parameter $\lambda$ (Fig. 1). This parameter controls the spatial correlation of rewards between options: the larger the parameter $\lambda$, the smaller the decay of correlation between more distant locations (options), yielding a flatter reward landscape (i.e., the mean rewards are more similar between two adjacent options). Using this model, we can estimate how much a participant subjectively believed that the rewards were spatially correlated. To distinguish from the true $\lambda$ used to generate the actual reward functions for each option/location in the environment, we denote the participant’s subjective belief about $\lambda$ as $\hat{\lambda }$. In other words, $\hat{\lambda }$ reflects the participant’s understanding of the generative rule for the environment.

We also assume that, after making each choice, the participant updates his/her understanding about the spatial distribution of rewards using the GP regression model^30^. That is, using $\hat{\lambda }$ and the experienced reward from the choice, the participant updates predictions about the mean and the standard deviation of each option in the environment in a Bayesian manner (see Supplementary Information for details).

**Figure 3:**
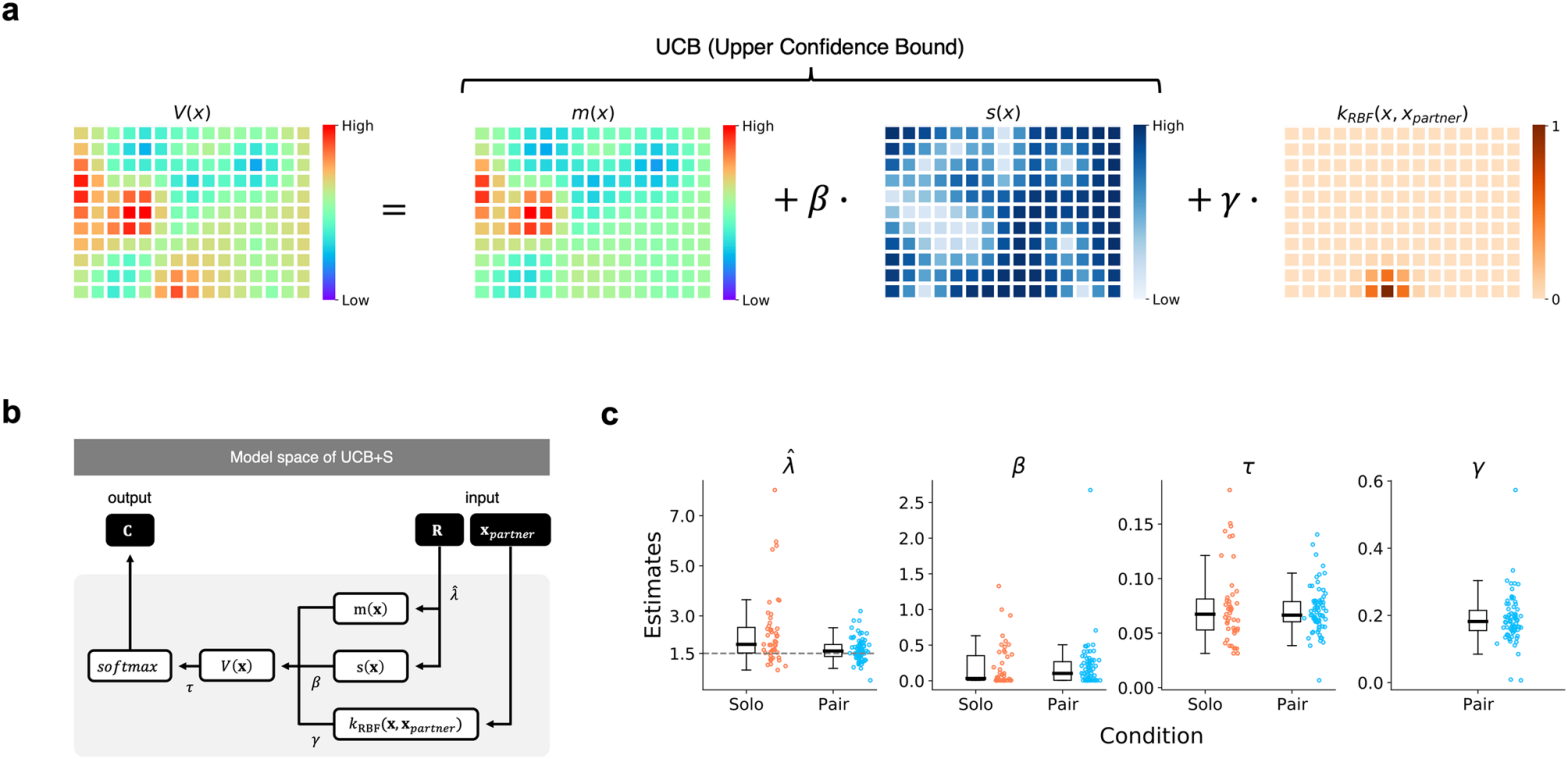
Overview of the computational model and estimated parameters. (**a**) Schematic illustration of the value function $V\left( {\mathbf{x}} \right)$ for options (${\mathbf{x}}$ is a vector representing the 165 options), when social learning is allowed (“UCB + S model” hereafter; see the text and Eq. (3) in Methods for explanation). Here, the value function $V\left( {\mathbf{x}} \right)$ is a weighted sum of the predicted mean $m\left( {\mathbf{x}} \right)$, the predicted variability $s\left( {\mathbf{x}} \right)$ (weighted by the uncertainty-premium parameter $\beta$), and the social information about the partner’s preceding choice $k_{RBF} \left( {{\mathbf{x}}, {\mathbf{x}}_{partner} } \right)$ (weighted by the imitation-bias parameter $\gamma$). (**b**) Model space of the UCB + S model. Based on Rewards $R$ from the current trial, a decision maker first updates predictions about the expected rewards $m\left( {\mathbf{x}} \right)$ and uncertainties $s\left( {\mathbf{x}} \right)$ for the whole environment by Gaussian process regression using the parameter $\hat{\lambda }$ (i.e., subjective belief about the generative rule). The decision maker then combines the information above into the overall value $V\left( {\mathbf{x}} \right)$. Here, in addition to the value derived from the UCB policy (sum of $m\left( {\mathbf{x}} \right)$ and $\beta \cdot s\left( {\mathbf{x}} \right)$, where $\beta$ is the uncertainty-premium parameter), the decision maker also incorporates the value $k_{RBF} \left( {{\mathbf{x}}, {\mathbf{x}}_{partner} } \right)$ accruing from social information about the partner’s preceding choice (see the text and Methods for details) weighted by his/ her imitation-bias parameter $\gamma$ (Fig. 3a). The overall values for the 165 options, $V\left( {\mathbf{x}} \right)$, are then transformed into a choice in the next trial. The participant’s choice $C$ is determined probabilistically according to the *softmax* function with the random exploration parameter $\tau$ (similar to temperature in reinforcement learning). (**c**) Estimates of the model parameters in the solo and pair conditions. For $\hat{\lambda }$, the true spatial correlation in the environment ($\lambda$ = 1.5) is shown as a dotted line.

The second element, the Upper Confidence Bound (UCB) policy, represents how a decision maker integrates several characteristics of an option into its value (i.e., decision utility; $V\left( {\mathbf{x}} \right)$ in Fig. 3a where ${\mathbf{x}}$ is a vector representing the 165 options). Most importantly, the UCB policy integrates the mean of each option (predicted mean $m\left( {\mathbf{x}} \right)$ in Fig. 3a) with its uncertainty (predicted standard deviation $s\left( {\mathbf{x}} \right)$ in Fig. 3a) to emphasize exploratory behavior under uncertainty. As shown in Fig. 3a, the values of options $V\left( {\mathbf{x}} \right)$ under the UCB policy are represented as $m\left( {\mathbf{x}} \right)$ + $\beta \cdot s\left( {\mathbf{x}} \right)$, where $\beta$ reflects the extent to which a decision maker systematically directs his/her explorations to more uncertain options^31–33^ (“uncertainty premium”).

For the model in the pair condition (UCB + S model), we further incorporated value $k_{RBF} \left( {{\mathbf{x}},{ }{\mathbf{x}}_{partner} } \right)$ accruing from social information into the option’s overall value (Fig. 3a). This component implies “imitation bias”, whereby a decision maker values options that are spatially closer to the option chosen by the partner in the preceding trial. The magnitude of imitation bias for a decision maker is governed by the parameter $\gamma$.

The overall value of options $V\left( {\mathbf{x}} \right)$, as defined above, are fed into the standard softmax function to probabilistically determine a participant’s choice behavior in the next trial. Similar to the temperature parameter in reinforcement learning^3^, we assumed another parameter $\tau$ that determines the decision maker’s degree of undirected exploration independent of value (i.e., “random exploration”). Thus, we have a total of four parameters in our model: $\hat{\lambda }$, $\beta$, $\gamma ,$ and $\tau$. Figure 3b provides a schematic summary of the UCB + S model (see Methods and Supplementary Information for details).

In the computational modeling, we conjecture that participants in the solo and pair conditions may adjust the parameter values of the model differently. In particular, if the accuracy of participants’ subjective beliefs about the generative rule for the reward landscape (Fig. 1) is improved by social learning opportunities in the pair condition, then the values of $\hat{\lambda }$ for spatial correlation would be more similar within actual pairs as compared to shuffled pairs (who did not interact with each other), and would also be closer to the true value $\lambda$ as compared to the solo condition.

### Modelling results

We evaluated the UCB + S (full) model quantitatively by comparing it with its sub-models using the Akaike Information Criterion^34^ (AIC). Because Wu and colleagues^20^ have already shown that the GP-UCB model provides a robust account of how solo learners utilize generalized beliefs across different environments, we took Gaussian process learning as given and tested the plausibility of the UCB + S model (i.e., the UCB model with the added component of imitation bias; Fig. 3a, b, and Eq. (3) in Methods). The alternative models that we tested are six sub-models of the UCB + S model (Supplementary Fig. S6). The model fitting revealed that the mean AIC of the UCB + S model was the smallest among the models tested (Supplementary Table S2). These results indicate that considering imitation bias in addition to the UCB policy^18–20,22,23^ provided reasonable overall fit to the participants’ choices in the pair condition. Figure 3c shows estimates of the four parameters of the UCB + S model.

Given the fit of the UCB + S model, we first explore the adaptive value of imitation bias ($\gamma$) on performance through a numeric simulation. In the experiment, we observed that the median of $\gamma$ was 0.18 (see Fig. 3c). How did this magnitude of imitation bias affect participants’ performance in the experiment? To answer this question, we varied the value of $\gamma$ systematically in the simulation while fixing the values of the other parameters ($\hat{\lambda }$, $\beta$, $\gamma ,$ and $\tau$) at their medians obtained from the experiment (see Supplementary Information for details; Supplementary Fig. S7). We observed that the effect of imitation bias on performance follows an inverted U-shape pattern: while a small to moderate imitation bias is beneficial, no bias or more extreme bias leads to less efficient performance. The observed magnitude of participants’ imitation bias (median $\gamma$ = 0.18) in the experiment seems to have contributed to the superior performance of the pair condition (see also Fig. 2a), as compared to the solo condition where $\gamma$ can be regarded as 0.

Next, we compare the parameter values estimated for the UCB + S model between the solo and pair conditions. There are three parameters that are directly comparable between the two conditions: (1) subjective belief about the generative rule $\hat{\lambda }$, (2) uncertainty premium $\beta$, and (3) random exploration $\tau$ (Fig. 3c). As seen in the figure, only the subjective belief parameter $\hat{\lambda }$ was significantly different between the two conditions (LMM: 95% CI [− 1.21, − 0.25], mean = − 0.72), while the two exploration parameters ($\beta$ for uncertainty-directed and $\tau$ for random exploration) were not (LMM: $\beta$: 95% CI [− 0.13, 0.12], mean = − 0.01; $\tau$: 95%CI [− 0.01, 0.01], mean = 0.00). It is noteworthy that $\hat{\lambda }$ was closer to the true value ($\lambda$ = 1.5) in the pair condition (median of $\hat{\lambda }$ = 1.59) than in the solo condition (median of $\hat{\lambda }$ = 1.86), implying that paired participants acquired a more accurate understanding of the spatial correlation of the environment.

**Figure 4:**
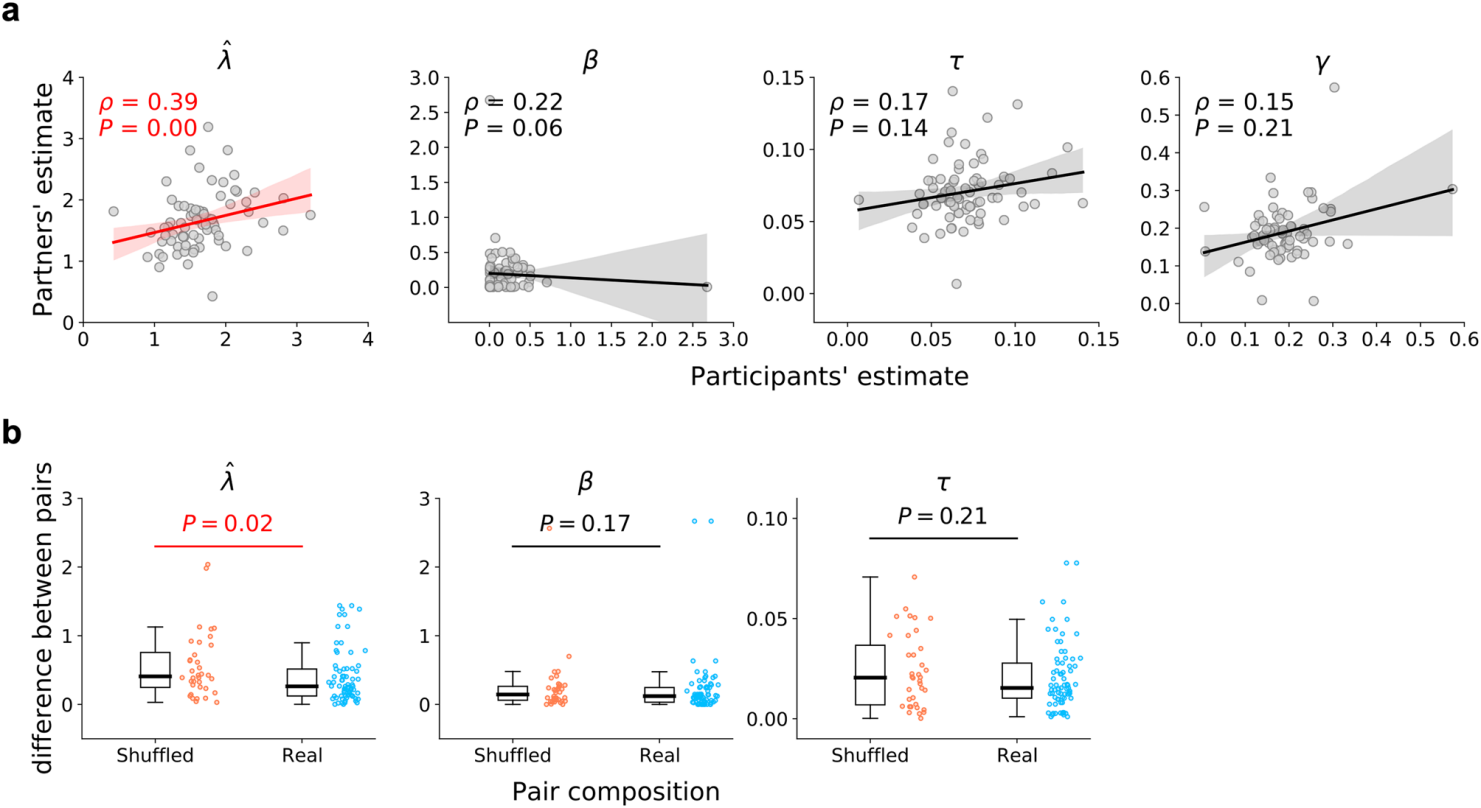
Paired participants were similar to each other in subjective understanding of the generative rule ($\hat{\user2{\lambda }})$, but not in the other parameters. (**a**) Correlation between paired participants’ parameter values. The solid lines and shaded areas represent linear regression and 95% credible intervals, respectively. (**b**) Parameter differences within pairs. Shuffled pairs (red) consist of two random participants in the pair condition, while real pairs (blue) consist of the actually paired participants.

At this point it remains unclear whether the observed improvement in the understanding of the generative rule ($\hat{\lambda })$ in the pair condition over the solo condition occurred independently for each individual within a pair or for both individuals at the pair level. To address this point, we conducted the following pair-level analyses. First, we examined correlations of parameter values between paired participants. As seen in Fig. 4a, among the four model parameters, only $\hat{\lambda }$ was significantly correlated between the paired participants (Spearman’s Rank Correlation test: $\rho$ = 0.39, *P* < 0.01), whereas the other three parameters were not ($\beta$: $\rho$ = 0.22, *P* = 0.06; $\tau$: $\rho$ = 0.17, *P* = 0.14; $\gamma$: $\rho$ = 0.15, *P* = 0.21). Second, to see if the parameter values converged within a pair due to social interaction, we created shuffled pairs (composed of two participants in the pair condition who were not actually paired) as a benchmark, and compared the absolute difference in parameter values for the real and shuffled pairs. As shown in Fig. 4b, again, the differences in $\hat{\lambda }$ were significantly smaller in the real pairs than in the shuffled pairs (Mann–Whitney U test: U = 1006.0, *P* = 0.02), while the uncertainty premium $\beta$ and the random exploration $\tau$ were not (Mann–Whitney U test: $\beta$: U = 1183.0, *P* = 0.17; $\tau$: U = 1204.0, *P* = 0.21). The same patterns were observed when compared with nominal pairs, in which two participants in the solo condition were randomly matched (Mann–Whitney U test: $\hat{\lambda }$: U = 398.0, *P* < 0.01; $\beta$: U = 683.0, *P* = 0.08; $\tau$: U = 734.0, *P* = 0.16; Supplementary Fig. S8).

## Discussion

Social learning is one of the core human capacities that have been vital in both growth and habitat expansion of human populations^28,35^. Previous research has shown that humans flexibly use various social learning strategies in response to the adaptive features of an environment^8,9,36^. Previous research has also used the exploration–exploitation tradeoff in information search^1–3^ as a common platform to clarify computational algorithms underlying social learning. Yet, as these works have been mainly concerned with how social information helps solve the exploration–exploitation tradeoff in an immediate environment (within-task learning^8,21,22,24^), it remains unknown whether and how social information may facilitate across-task learning, whereby acquired knowledge in one environment is generalized or extended to other related environments. Given the exponential expansion of possible search spaces in the modern world, it is fundamental to study how such across-task learning^17–20^ may emerge as a form of collective intelligence through social interaction. Here, we investigated this issue through a laboratory experiment and accompanying computational modeling that focused on social learning when participants engaged in a series of foraging sessions in spatially-correlated reward landscapes that were generated according to a common rule (Fig. 1).

This experiment has revealed the following observations. Firstly, the paired participants consistently outperformed the solo participants in all six spatially-correlated 165-armed bandit environments (Fig. 2a, b), replicating the performance improvement due to social learning in simpler MAB environments without spatial correlations^7^. More central to our argument, the superiority of the paired participants to the solo participants was more pronounced in later sessions (Fig. 2c). This suggests that the paired participants may have developed some generalizable knowledge^10,11^ across the six environments through social interaction. This conjecture was also consistent with the results of participants’ exploration behavior in terms of unique choices that they made during information search (Fig. 2d,e). As the sessions progressed, the paired participants switched to exploitation in earlier phases compared to the solo participants, suggesting that they had acquired better insights about the common feature of the reward landscapes (see also Supplementary Figs. S4 & S5).

To explore this conjecture further, we next analyzed each participant’s choice behavior using a computational model. The model comparison revealed that the full model (UCB + S model; see Fig. 3a,b and Supplementary Fig. S6) was the best in terms of AIC (Supplementary Table S2), and it also passed the parameter recovery test (Supplementary Figs. S9 & S10). This model assumed that participants assessed the value of an option as a weighted sum of the predicted mean $m\left( {\mathbf{x}} \right)$, the predicted variability $s\left( {\mathbf{x}} \right)$ (weighted by the uncertainty-premium parameter, $\beta$), and the social information about the partner’s preceding choice $k_{RBF} \left( {{\mathbf{x}},{ }{\mathbf{x}}_{partner} } \right)$ (weighted by the imitation-bias parameter, $\gamma$). It is important to note the presence of this latter parameter in the selected UCB + S model, by which participants incorporate social information into valuation of the choice options. This model-selection result is consistent with previous research on simple imitation learning, showing that people tend to copy others’ choices in a shared environment^21,22^. The numeric simulation with the UCB + S model also revealed that the effect of imitation bias on performance follows an inverted U-shape pattern: while a small to moderate imitation bias (which was actually observed in the behavioral experiment with median $\gamma$ = 0.18) is beneficial, no bias or more extreme bias is detrimental to performance (Supplementary Fig. S7).

However, we should emphasize that the success of such simple copying of other’s choices in solving the exploration–exploitation tradeoff in one environment does not necessarily mean that current knowledge has been acquired at a general level (e.g., a common generative rule) that can be extended to other related environments. In our model, such understanding about the common generative rule is captured by $\hat{\lambda }$, which reflects the participant’s understanding of the spatially-correlated feature of the environment. Among the parameters of the UCB + S model, $\hat{\lambda }$ was the only parameter that improved significantly in the pair condition (i.e., was closer to the true environmental $\lambda$ = 1.5) over the solo condition (Fig. 3c), suggesting that paired participants had acquired some insights about the common rule underlying the reward landscapes.

Furthermore, among the four parameters, $\hat{\lambda }$ was again the only parameter that was correlated (Fig. 4a) and converged significantly within real pairs (as compared to the shuffled pairs: Fig. 4b; and to the nominal pairs: Supplementary Fig. S8). This result indicates that the social interaction between decision makers played an essential role in developing insights about the generative rule. In other words, across-task learning may be achieved as a kind of collective intelligence, emerging through reciprocal interactions of learning with a partner, rather than one-way observational learning^37–39^.

There are several limitations in our study that should be addressed in future research. Firstly, while our results showed that social interaction opportunities facilitated across-task learning by refining participants’ understanding of the generative rule, the identity of the critical interaction element in this process remains unknown. There are several dimensions that we believe warrant future investigation. For instance, the amounts or kinds of information exchanged during interaction may be critical. In this experiment, we informed the paired participants only of the partner’s behavioral choice in the preceding round. In the real world, however, people sometimes have access to the actual outcomes (e.g., the rewards) from others’ choices as well (see Goldstone, Ashpole, and Roberts (2005)^27^ for effects of visibility/invisibility of choices/rewards of other individuals under competitive group foraging). It is important to examine how and when the amounts/kinds of social information may facilitate across-task learning (e.g., is more information or less information better for understanding of the generative rule^7^; how do people incorporate the history of others’ behavioral choices in their own valuation of the options^29^). We also conjecture that the emergence of division of labor via coordination of collective search^40,41^ may be of key importance. As we observed in the experiment, individuals exhibit various search strategies, psychological propensities for directed or random exploration, risk tolerance, and so on under the exploration–exploitation tradeoff. When grouped, people can potentially capitalize on such individual differences for efficient information search^42,43^, which may facilitate across-task learning in multiple related environments. To test this conjecture, we will need to develop a fine-grained computational model of trial-by-trial interaction processes and test its validity by manipulating the social process experimentally, for example by introducing artificial “bots” as interaction partners^39,44^. Future research incorporating these techniques seems promising for illuminating the interaction elements that are critical for across-task learning in groups.

Secondly, the key environmental parameter, $\lambda$, that generated each search space was fixed to be the same (= 1.5) across the sessions in this experiment. Thus, our results remain as a demonstration with the specific spatial correlation employed in the experiment. To see whether our observations may be robust across broader situations, future research should vary the value of $\lambda$ systematically and test the hypothesis across different values^18–20^. Relatedly, we estimated a participant’s subjective understanding about the generative rule, $\hat{\lambda }$, by collapsing across all six sessions. This analysis precluded seeing how $\hat{\lambda }$ may have evolved across the six sessions. We actually tried to estimate $\hat{\lambda }$ separately for the first half (sessions 1–3) and the second half (sessions 4–6), but neither parameter estimation converged due to the small number of samples (25 × 3 = 75 trials). In future work, increasing the number of trials per session would be desirable to address this point, although this may introduce other problems such as boredom or habituation among participants.

Finally, introducing other environmental structures will be important to further test the possibility of across-task learning through social interaction. Here we have used a spatially-correlated structure. On the other hand, some previous research on within-task learning through social interaction has used temporally-correlated structure (a “restless” MAB), whereby mean values of options are autocorrelated over time (i.e., more similarity between nearer time points^24,45^). Comparing the spatially-correlated and temporally-correlated structures for emergence of across-task learning may provide a more comprehensive picture about its robustness and/or boundary conditions.

In this article, we have shown that social learning through real interaction can facilitate across-task learning in a large search space by improving the participant’s understanding of a common generative rule across environments. To our knowledge, this is the first study that has demonstrated the empirical relation between social learning opportunities and across-task learning. In the social learning literature, it has repeatedly been shown that social information is actively used through various social learning strategies to promote within-task learning about the immediate environment^21,22^. However, almost no research has addressed whether and how the use of social information may promote higher-level understanding about generative rules to enable across-task learning^28^. We believe that investigating how collective intelligence can emerge at a higher level beyond the immediate task environment is critical for modern societies, where decision makers can be overwhelmed by large amounts of locally useful yet fragmentary information.

## Methods

### Participants

Participants (n = 121; 61 female; mean ± s.d. age: 21.4 ± 0.2 years) were recruited from the subject pools of Hokkaido University (Hokkaido, Japan) and the University of Tokyo (Tokyo, Japan). For about 70 min of participation, they were paid 1,479 yen (s.d.: 289.58 yen) on average (about 13 ± 2.5 USD), including an additional performance bonus. The experiment was approved by the Ethics Committee of Hokkaido University and that of the University of Tokyo, in accordance with the Declaration of Helsinki. Written informed consent was obtained from each participant before the experiment, and no deception was involved. Participants were instructed to collect as many points as possible to increase their rewards. Three participants were not able to participate in the last three sessions due to failures of the computer system; thus only the data from their first three sessions were included in the analysis.

### Design & task

Participants were randomly assigned to the solo condition (*n* = 47) or the pair condition (*n* = 74). Upon arrival at the laboratory, each participant was seated in a private soundproof cubicle and was not able to communicate with others.

In the experiments, they worked on a multi-armed bandit (MAB) task with 165 options. As seen in Fig. 1, the 165 options in the search space were arrayed in a 11 × 15 grid on a computer screen. All participants first completed a 10-trial practice session individually, followed by the main sessions. Participants worked on a total of six unique environments, the order of which were randomized across participants (see Fig. 1 caption for details). Participants made 25 choices (i.e., 25 trials) for each environment, and afterwards were asked to predict reward values for 16 options, which were randomly selected from the set of unchosen options in the environment. Of the 16 options, eight were sampled from the higher-reward area in which the reward was equal to or higher than 0.5 in the min–max normalization scale, and the other eight were sampled from the lower-reward area (see Supplementary Fig. S11). As a measure of the accuracy of subjective estimates, we calculated the mean absolute deviation of the participant’s estimates from the true reward values.

### Procedure

Before starting, participants were explicitly informed that the expected rewards from “boxes” in the environment would be spatially correlated (i.e., rewards from more nearby boxes would be more similar), but the exact magnitude of the correlation (i.e., the generative rule) was not specified. We generated six environments in advance from an RBF kernel (see Eq. (1)) with $\lambda$ = 1.5. In the main sessions, participants worked on these six environments in a randomized order (Fig. 1). For each of the 25 trials under each environment, participants chose one of the 165 boxes and received a point reward. The point reward was randomly generated from a distribution unique to the box with normally distributed noise, $\varepsilon \sim N\left( {0,1} \right)$. The point rewards from the chosen boxes were kept visible until the end of the 25 trials. If the same box was chosen several times, the reward display was updated with the most recent reward. For each of the six environments, we rescaled point-reward values to range from 0 to a maximum value randomly generated from a uniform distribution [80, 120]. Thus, the value of the globally optimal box was different across the six environments, so that participants could not guess the value from previous experience in another environment. In the pair condition, participants could observe the location of the box chosen by the partner in the preceding trial, but not the reward value obtained by the partner.

### Statistical analyses

We introduced generalized linear mixed models (GLMMs) in the following three analyses. First, to analyze how the solo individuals and the pairs performed over the course of sessions and trials (Fig. 2), we used a hierarchical Bayesian Gaussian regression model with a random effect of person (participant ID). The dependent variable was the reward value obtained from each choice. The model included fixed effects of experimental condition (0: solo, 1: pair), session (standardized), trial (standardized), and all two-way interactions between the fixed effects (Supplementary Table S1).

Second, to test whether the experimental condition and session progress affected the proportions of unique choices in the first, second, and third stages of the task, we used a hierarchical Bayesian binomial regression model with a random effect of person. The dependent variable was the number of unique choices a participant made, and fixed effects were the condition (0: solo, 1: pair), session (standardized), and all two-way interactions between the fixed effects.

### Computational models

We used the GP-UCB model^30^, with an added component to analyze the effects of social learning. The model assumes that, through Gaussian process regression, participants update their current knowledge of the spatial distribution of rewards; they make predictions about the expected mean $m\left( {\mathbf{x}} \right)$ and the associated uncertainty $s\left( {\mathbf{x}} \right)$ (estimated as a standard deviation) for each option x, conditioned on the previous observations of rewards ${\mathbf{y}}_{t} = \left[ {y_{1} ,{ }y_{2} , \ldots ,y_{t} } \right]^{T}$ at inputs ${\mathbf{X}}_{t} = \left[ {{\mathbf{x}}_{1} ,{\mathbf{x}}_{2} , \ldots ,{\mathbf{x}}_{t} } \right]^{ }$. The estimates about $m\left( {\mathbf{x}} \right)$ and $s\left( {\mathbf{x}} \right)$ at trial t are provided in the form of the posterior distribution of the 165-dimensional Gaussian distribution and are updated trial-by-trial according to the Bayes rule. The covariance between options ${\mathbf{x}}$ and ${\mathbf{x}}^{{\prime }}$ in the Gaussian process prior are set by using a radial basis function (RBF) kernel $k_{RBF} \left( {{\mathbf{x}},{ }{\mathbf{x}}^{{\prime }} } \right)$ as below:

1 $$k_{RBF} \left( {{\mathbf{x}},{\mathbf{x}}^{{\prime }} } \right) = {\text{exp}}\left( { - \frac{{\left\| {{\mathbf{x}} - {\mathbf{x}}^{{\prime }} } \right\|^{2} }}{\lambda }} \right),$$

where the length-scale parameter $\lambda$ (> 0) controls how quickly the spatial correlation of rewards between options decay towards zero. A smaller $\lambda$ means a more rapid decay in correlation with increase in distance between two options. To distinguish from the true $\lambda$ used to generate the actual reward function of the environment, we denote the participant’s subjective belief about $\lambda$ as $\hat{\lambda }$. Hereafter, $\lambda$ is used to denote the objective rule for generating the environment, and $\hat{\lambda }$ is used to denote the subjects’ belief estimated by the model fitting. We regard the parameter $\hat{\lambda }$ as a participant’s understanding about the generative rule for the environment.

UCB sampling provides the value (utility) function of options at each trial, based on estimates about mean rewards $m\left( {\mathbf{x}} \right)$ and the underlying uncertainty $s\left( {\mathbf{x}} \right)$ from Gaussian process regression:

2 $${\text{UCB}}\left( {\mathbf{x}} \right) = m\left( {\mathbf{x}} \right) + \beta \cdot s\left( {\mathbf{x}} \right),$$

where $\beta { }( > 0)$ is a free parameter governing how much uncertainty is valued, relative to capitalizing on expectations of reward. Therefore, $\beta$ can be interpreted as the uncertainty premium. Furthermore, to consider the effects of social information in the pair condition (see UCB + S model in Fig. 3a,b), we added value accruing from imitation to the UCB value, producing an overall valuation function $V\left( {\mathbf{x}} \right)$:

3 $$V\left( {\mathbf{x}} \right) = {\text{UCB}}\left( {\mathbf{x}} \right) + \gamma \cdot k_{RBF} \left( {{\mathbf{x}}, {\mathbf{x}}_{partner} } \right),$$

where $\gamma$ is a free parameter governing the magnitude of imitation bias (Fig. 3a), and ${\mathbf{x}}_{partner}$ is the partner’s choice in the preceding trial. Because social learning opportunities only informed the participant about the location of the partner’s preceding choice and not the actual reward value obtained by the partner (i.e., a ‘frequency-dependent’ rather than ‘payoff-based’ social learning strategy^21^), we assumed in Eq. (3) that the vicarious reward value of the partner’s preceding choice is 1^22^ and those of neighboring options decay according to the distance from the partner’s choice, denoted by $k_{RBF} \left( {{\mathbf{x}},{ }{\mathbf{x}}_{partner} } \right)$ ($0 < k_{RBF} \left( {{\mathbf{x}},{ }{\mathbf{x}}_{partner} } \right) \le 1$).

The overall valuation function was combined according to a softmax choice rule, producing choice probabilities of each option at each trial:

4 $$P\left( x \right) = \frac{{{\text{exp}}\left( {V\left( {\mathbf{x}} \right)/\tau } \right)}}{{\mathop \sum \nolimits_{j = 1}^{165} {\text{exp}}\left( {V\left( {{\mathbf{x}}_{j} } \right)/\tau } \right)}},$$

where $\tau { }( > 0)$ is a free parameter governing the softmax temperature. $\tau$ reflects random exploration. We call this model the UCB + S model (Fig. 3a,b). By a series of simulations prior to the experiment, we confirmed that the four free parameters of the UCB + S model (i.e., subjective belief about the generative rule $\hat{\lambda }$, uncertainty premium $\beta$, random exploration $\tau$, and the imitation bias $\gamma$) are distinguishable from each other and robustly recoverable (see the section on parameter recovery below).

### Model fitting procedure

Each model was fitted separately to the behavioral data of each participant. We used maximum likelihood estimation (MLE) with a Differential Evolution algorithm^46,47^ to estimate free parameters. From the log-likelihood, we derived the Akaike Information Criterion^34^ (AIC) defined as

5 $$AIC = - 2LogLik + 2k$$

where $k$ is the number of free parameters. All fitting procedures were implemented using a set of packages in Python (v3.7.6). We used Scipy.optimize (v1.5.2) for MLE, and scikit-learn (v0.23.2) for Gaussian process regression.

### Parameter recovery and comparison between parameters

To ensure that each parameter in the selected model (UCB + S model) robustly captured separate and distinct phenomena, we conducted a parameter recovery test^48^ (see Supplementary Information). As seen in Supplementary Fig. S9, for all free parameters, the generated and the recovered parameter estimates were highly correlated. Also, as seen in Supplementary Fig. S10, the off-diagonal elements of the correlation matrix were poorly correlated (at most *r* = 0.12), indicating that the free parameters in the UCB + S model were independently estimated from each other in the fitting procedure. The good parameter discovery displayed by the winning model indicated that their estimates are meaningful and can be statistically compared. As assumptions about normal distributions were not verified, we used nonparametric tests for comparing model parameters. A LMM with a random effect of pair was used for comparing parameters between the solo and pair conditions.

## Supplementary Information

Supplementary Information.

## Data Availability

Anonymized participant data, model simulation data, and the codes used for all models and analyses are available at https://osf.io/9vayd/.
