# Supplementary material for: Insights about the common generative rule underlying an information foraging task can be facilitated via collective search
Source: Sci Rep. 2022 May 16;12:8047. doi: 10.1038/s41598-022-12126-3 (PMC9110753; doi:10.1038/s41598-022-12126-3)
Supplement: Supplementary file 1 — Supplementary Information. [file 41598_2022_12126_MOESM1_ESM.pdf]

Supplementary Information

Insights about the common generative rule underlying an information foraging task can be facilitated via collective search

Aoi Naito[1,2], Kentaro Katahira[3] & Tatsuya Kameda[1,4,5*]

[1]Department of Social Psychology, The University of Tokyo, Tokyo 113-0033, Japan

[2]Japan Society for the Promotion of Science, Tokyo 102-0083, Japan

[3]Human Informatics and Interaction Research Institute, National Institute of Advanced Industrial Science and Technology (AIST), Tsukuba 305-8566, Japan

[4]Brain Science Institute, Tamagawa University, Tokyo 194-8610, Japan

[5]Center for Experimental Research in Social Sciences, Hokkaido University, Sapporo 060-0810, Japan

[*]Corresponding author.

Email: tkameda@l.u-tokyo.ac.jp (T. Kameda).

Experimental procedure

Participant recruitment

Participants were recruited from subject pools of Hokkaido University and the University of Tokyo, consisting of undergraduates and graduate students in each university. Upon arriving at the laboratory, participants signed a consent form and viewed instructions via the computer screen. The data were collected over a 5-day period at Hokkaido University (from August 16, 2019 to August 22, 2019; weekdays only) and an 8-day period at the University of Tokyo (from September 17, 2019 to September 27, 2019).

Stimuli & materials

Stimuli were presented on a 23.8-inch EIZO FlexScan EV2451 with a spatial resolution of 1920 × 1080 pixels. Experiments were programmed in PsychoPy[1] (v1.90.3) and data analyses were performed using custom software written in Python.

Statistical analysis

All statistical analyses were implemented using a set of packages in Python (v3.7.6), including NumPy (v1.10.1) and Pandas (v1.0.1) for preprocessing data, SciPy (v1.5.2) for statistical calculations[2], and Matplotlib (v3.1.3) for drawing figures. To perform MCMC sampling, we used PyStan[3] (v2.19.0.0).

Supplementary results

Probability of finding the optimal option

We define the "box" with the highest reward in each environment as the optimal option. As seen in Supplementary Fig. S2, the mean proportion of sessions (out of 6) during which participants found the optimal option was slightly higher in the pair condition than in the solo condition (solo: 0.43 / pair: 0.48). We analyzed the proportions of optimal option discovery using a binomial GLM with a fixed effect of condition as a dummy variable (solo: 0 / pair: 1). The model was fit using MCMC, and the 95% Bayesian credible interval ([-0.06, 0.52]) crossed 0, indicating that the difference in proportion of optimal option discovery was not significant between the two conditions.

Exploration pattern in terms of "migration" length

We examined the mean migration length from trial t-1 to that at trial t as another measure of participants' exploration behaviors (Supplementary Fig. S4). We measured migration length using Manhattan (i.e., "city block") distance. As seen in the figure, for both the solo and pair conditions, participants initially migrated long distances ("exploration") but tended to gradually settle in and capitalize on a local patch ("exploitation"). The median of the migration length in the pair condition (=3.08) was smaller than that in the solo condition (=3.27). However, there was no significant difference between the two conditions ($t(117)=-1.67$, $p = 0.1$, $d=-0.32$, CI: -0.94 to 0.14; Supplementary Fig. S4, left). Also, even when decomposing the mean migration length data into the first (trials 1 to 8), second (trials 9 to 16), and third (trials 17 to 25) phases, we found no significant difference between conditions in any phase (Kolmogorov-Smirnov test: first: $D=0.13$, $p=0.69$; second: $D=0.19$, $p=0.22$; third: $D=0.17$, $p=0.34$; Supplementary Fig. S4, right).

Subjective estimates about unchosen options

As a probe for participants' understanding of the environmental structure, we asked each participant to provide subjective estimates for the (unknown) rewards from options that she/he did not choose in each session. At the end of each session, participants reported their estimates for 16 options, which were randomly selected from the set of unchosen options. Half of these 16 options had higher rewards (equal to or higher than 0.5 in the min-max normalization scale) and the other half had lower rewards (Supplementary Fig. S11). As a measure of the accuracy of subjective estimates, we calculated the mean absolute deviation of the participant's estimates from the true reward values. The results are shown in Supplementary Fig. S5.

Although the effect is weak (see Supplementary Table S3), subjective estimates for the unchosen options tended to be more accurate in the pair condition than in the solo condition (LMM, condition: 95%CI: [-1.86, 0.26], mean=-0.76). This effect also tended to be more pronounced in the higher-reward areas than in the lower-reward areas (LMM, condition × option-type: 95% CI: [-4.52, 0.46], mean=-2.03). Although statistically not strong, these cognitive patterns are in line with the behavioral results (e.g., Fig. 2c, 2d, and 2e; Supplementary Fig. S4) indicating that social learning opportunities helped participants learn the common environmental structure.

Modeling

Gaussian process regression

A Gaussian process is defined as:

$$f \sim \mathrm{GP}(m(\mathbf{x}), \mathbf{K}), \tag{1}$$

where real-valued scalar output $\mathbf{f} = (f(\mathbf{x}_1), f(\mathbf{x}_2), \ldots, f(\mathbf{x}_N))$ follows a Gaussian distribution with mean $m(\mathbf{x})$ and covariance matrix $\mathbf{K}$, the elements of which are denoted by $\mathbf{K}_{n,n'} = k(\mathbf{x}_n, \mathbf{x}_{n'})$, given input: $[\mathbf{x}_1, \mathbf{x}_2, \ldots, \mathbf{x}_N]$.

As described in Methods, we used a Radial Basis Function (RBF) kernel $k_{RBF}(\mathbf{x}, \mathbf{x}')$ to express the covariance:

$$k_{RBF}(\mathbf{x}, \mathbf{x}') = \exp\left(-\frac{\|\mathbf{x} - \mathbf{x}'\|^2}{\lambda}\right), \tag{2}$$

where the length-scale parameter $\lambda\ (> 0)$ determines how quickly correlations between options $\mathbf{x}$ and $\mathbf{x}'$ decay towards zero: The smaller the λ, the more rapidly correlations between options decay with the increase in distance between the options.

We fixed the prior mean to the expected mean of payoffs, $m(\mathbf{x}) = 0.5$, after rescaling by min-max normalization. Conditioned on the observed data $D_t = \{\mathbf{x}_j, y_j\}_{j=1}^{t}$, where $y_i \sim N(f(\mathbf{x}_j), \sigma^2)$ with $\sigma = 0.01$, the posterior distribution at position $\mathbf{x}$ obeys a Gaussian distribution, and is analytically given according to the Bayes rule:

$$p(y^*|\mathbf{x}^*, \mathcal{D}_t) = \mathcal{N}(\mathbf{k}_*^T(\mathbf{K} + \sigma^2\mathbf{I})^{-1}\mathbf{y}_t, k(\mathbf{x}_*, \mathbf{x}_*) - \mathbf{k}_*^T(\mathbf{K} + \sigma^2\mathbf{I})^{-1}\mathbf{k}_*), \tag{3}$$

where $\mathbf{y}_t = [y_1, y_2, \ldots, y_t]^T$ and $\mathbf{k}_* = [k(\mathbf{x}_1, \mathbf{x}_*),\ k(\mathbf{x}_2, \mathbf{x}_*), \ldots, k(\mathbf{x}_t, \mathbf{x}_*)]$ is the covariance between each observed input and the new input $\mathbf{x}_*$.

Alternative models

In Supplementary Fig. S6, we show six variations of models in addition to the UCB+S model. All alternative models are nested in terms of the sampling policy[4]. As described in the main text, we express the sampling policy of the UCB+S model by the linear combinations of the utilities of expected rewards $m(\mathbf{x})$, the uncertainty premium $\beta \cdot s(\mathbf{x})$, and the imitation bias $\gamma \cdot k_{RBF}(\mathbf{x}, \mathbf{x}_{partner})$. The alternative models are represented by partial combinations of these elements. The model without the uncertainty premium $\beta \cdot s(\mathbf{x})$ and the imitation bias $\gamma \cdot k_{RBF}(\mathbf{x}, \mathbf{x}_{partner})$ was described as "Pure-Exploit". The model without the term of expected rewards $m(\mathbf{x})$ and the imitation bias $\gamma \cdot k_{RBF}(\mathbf{x}, \mathbf{x}_{partner})$ was described as 'Pure-Explore'. The other three models containing the imitation bias $\gamma \cdot k_{RBF}(\mathbf{x}, \mathbf{x}_{partner})$ were described as "UCB+S", "Pure-Exploit+S", or "Pure-Explore+S". The model omitting all of the above elements was described as 'Random' because there is no value-based term in the model (i.e., all options are chosen from a uniform distribution with probability of 1/165). See Supplementary Table S2 for mean AICs of these models.

Parameter recovery

We checked the performance of parameter recovery for the UCB+S model by generating 100 artificial datasets with random values for the four parameters. As seen in Supplementary Fig. S9, for all four parameters, correlations between individual true parameters and recovered parameters were high and positive (λ: r=0.98, β: r=0.86, τ: r=0.93, γ: r=0.90). We also confirmed that the recovery process yielded no correlations between the four recovered parameters (the correlations between the recovered parameters were all slight and negative; see Supplementary Fig. S10).

Numeric simulation of the effect of imitation bias on performance

We explored how task performance may change as a function of the magnitude of the imitation bias (γ). In this simulation, we assumed that the agents made choices according to the UCB+S model in the same task setup as in the experiment. We ran a total of 1,000 simulations for each magnitude (γ=0, 0.1, 0.2, 0.3, 0.4, 0.5, or 0.6) of the imitation bias, while fixing the other three parameters at the medians of the estimated values across the solo and pair conditions (λ= 1.66, β=0.07, τ=0.07). The two agents in a pair had the same parameter values.

We observed that the effect of imitation bias follows an inverted U-shape pattern. While a small to moderate imitation bias (e.g., γ=0.3) is beneficial, more extreme bias (γ=0.6) or no bias (γ=0) leads to less efficient performance (Supplementary Fig. S7, left). Recall that most participants in the experiment had a small to moderate imitation bias (median γ=0.18, Fig. 3c). This magnitude of imitation bias seems to have contributed to the superior performance of the pair condition, as compared to the solo condition (Fig. 2a; see also Supplementary Fig. S7).

Supplementary Figures

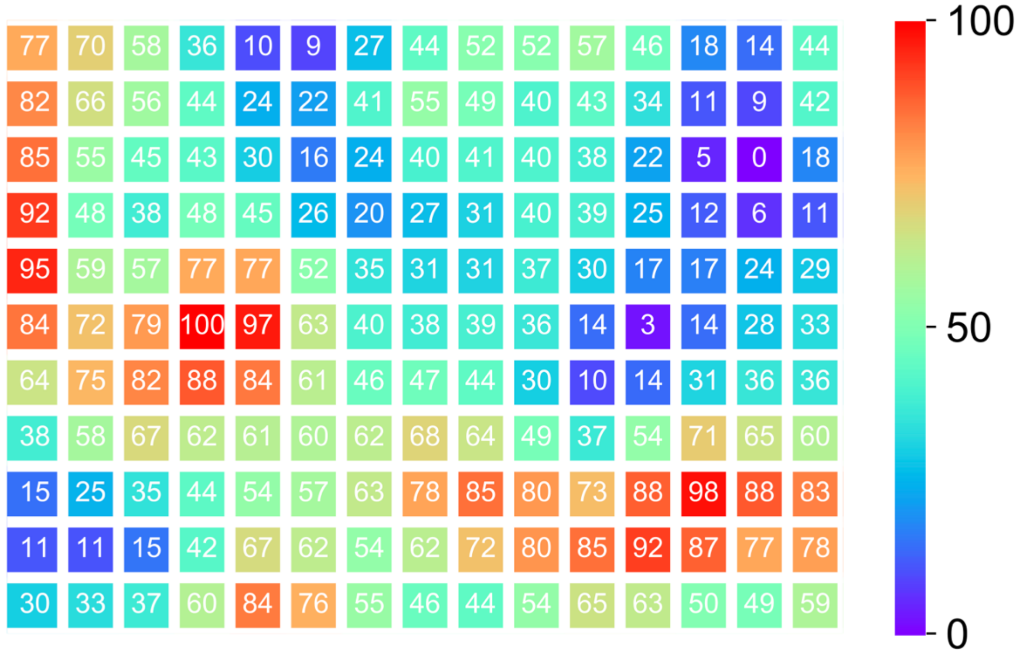

Fig. S1 | Search environment used in the practice session. All participants worked on the same reward landscape individually in the practice session. This environment was sampled from an identical Gaussian process prior with λ=1.5, but was different from those used in the six main sessions.

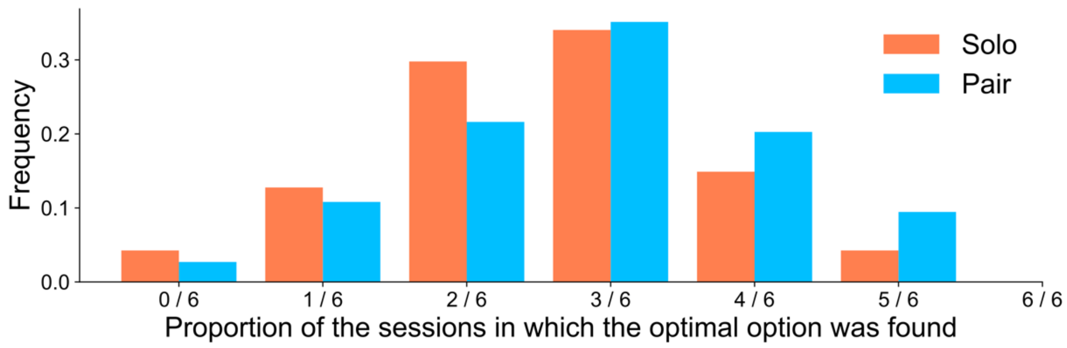

Fig. S2 | Distributions of participants who found the optimal (most rewarding) option in *n* out of 6 sessions in the solo (red) and pair (blue) conditions. For the three participants who were not able to participate in the last three sessions due to failures of the computer system (see Methods), we realigned their data (out of 3) to accord with the 0/6 to 6/6 scale. See "probability of finding the optimal option" in the Supplementary Results section for statistical results.

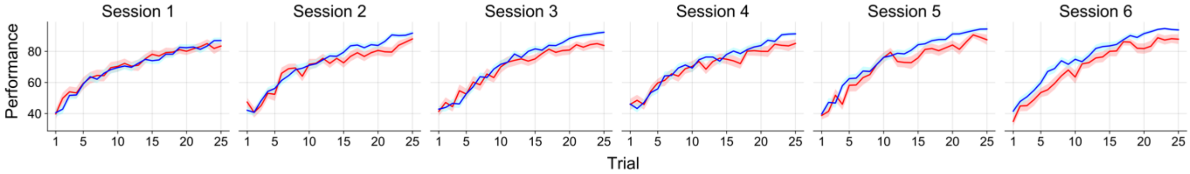

Fig. S3 | Session-by-session transitions of learning curves over the 25 trials in the solo (red) and the pair conditions (blue). For each session, performances were averaged across the six environments.

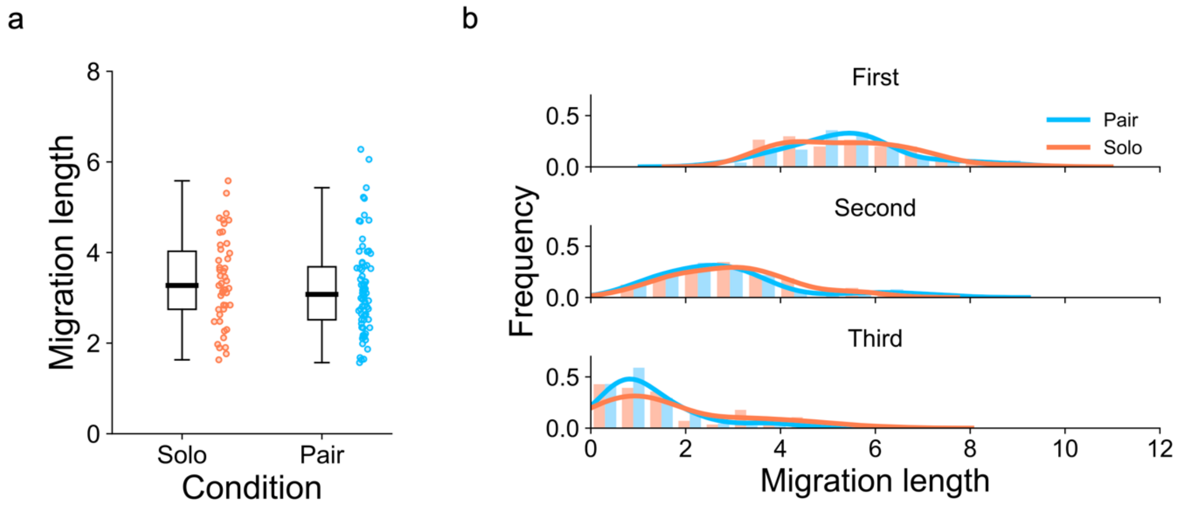

Fig. S4 | Comparison of migration length between conditions. (a) Mean migration length from trial *t*-1 to trial *t* in terms of Manhattan distance. Each dot represents one participant. **(b)** Histograms and Kernel Density Estimation plots of the mean migration length in the first (trial 1 to 8), second (trial 9 to 16), and third (trial 17 to 25) phase of each session. See "exploration pattern in terms of migration length" in the Supplementary Results section for statistical results.

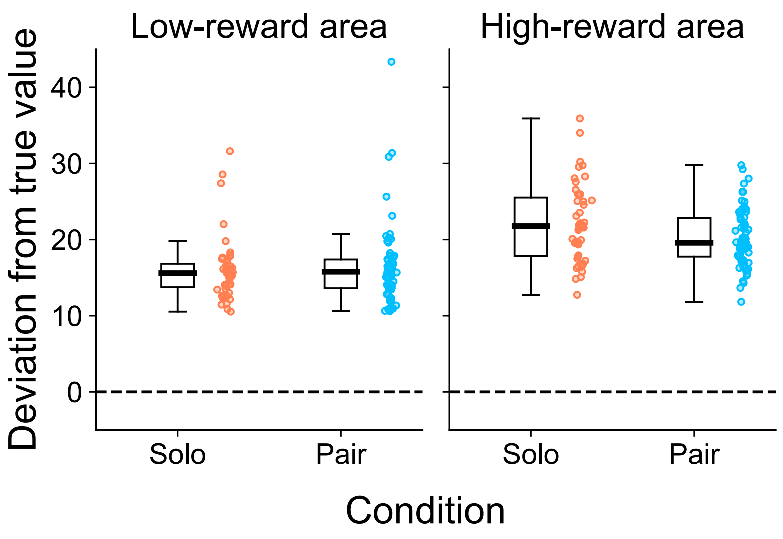

Fig. S5 | Accuracy of participants' subjective estimates about unchosen options. The mean absolute deviations of estimates from the true values of unchosen options are shown separately for the lower-reward area (left) and the higher-reward area (right). Each dot represents one participant's mean absolute deviation. Zero (dashed line) corresponds to perfectly accurate estimation, and larger values indicate less accurate estimates. See "subjective estimates about unchosen options" in the Supplementary Results section for details.

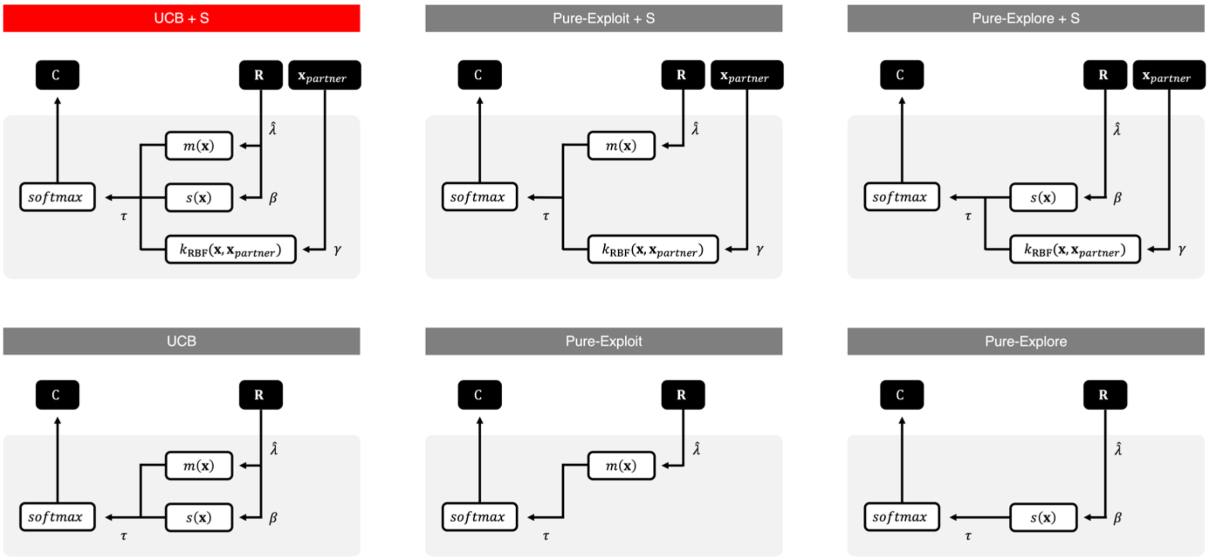

Fig. S6 | Model space: UCB+S: The UCB+S model is our target model, which has four free parameters in total. Rewards R are used to estimate the expected rewards $m(\mathbf{x})$ and uncertainties $s(\mathbf{x})$ in the whole search space using Gaussian Process regression with a length-scale $\hat{\lambda}$. In the process of forming the valuation function of each option, both UCB (the sum of $m(\mathbf{x})$ and $\beta \cdot s(\mathbf{x})$) and the social information (derived from the option chosen by the partner $\mathbf{x}_{partner}$ and an RBF kernel function $k_{RBF}(\mathbf{x}, \mathbf{x}_{partner})$) influence the learning process. Participants' choices c are sampled according to the *softmax* function with a temperature τ. Pure-Exploit+S: In the process of forming the valuation function of each option, the expected uncertainties $s(\mathbf{x})$ of options have no effect on the learning process. Thus, participants are assumed to exploit their own information during learning, but not actively explore (i.e., they 'purely exploit' the already-known information). Pure-Explore+S: In the process of forming the valuation function of each option, the expected rewards $m(\mathbf{x})$ of options have no effect on the learning process. Thus, participants are assumed to explore the not-yet-observed options during learning, but not actively exploit their own information (i.e., they 'purely explore' the not-yet-observed information). UCB, Pure-Exploit, and Pure-Explore are the sub-models of the UCB+S model, the Pure-Exploit+S model, and the Pure-Explore+S models, respectively, removing the social information. That

is, these models assume that, in the process of forming the valuation function of each option, the social information $\mathbf{x}_{partner}$ has no effect on the learning process. Thus, participants behave as if they are learning individually, even though the partner's information is available.

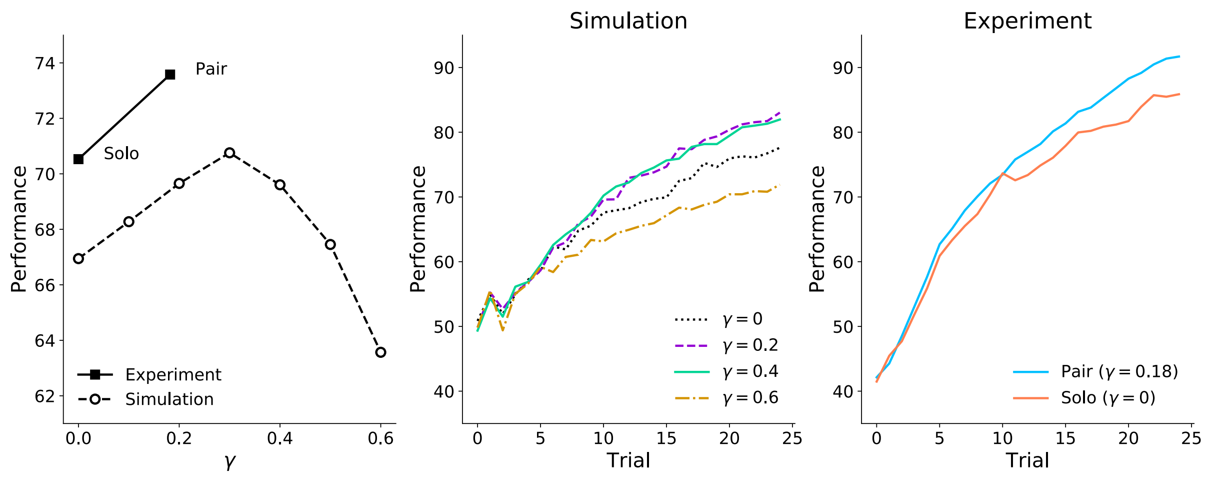

Fig. S7 | The effect of imitation bias (γ) on performance. (Left) Comparison between the results of the numeric simulation (dashed line) and the behavioral results from the experiment (solid line) in terms of mean performance. Note that the effect of imitation bias follows an inverted U-shape pattern: while a small to moderate imitation bias (e.g., γ=0.3) is beneficial, no bias (γ=0.0) or more extreme bias (γ=0.6) leads to less efficient performance. **(Center)** Simulation results showing the mean performance of agents over 25 trials with γ=0, 0.2, 0.4, 0.6. **(Right)** Behavioral results showing the mean performance of participants over 25 trials in the solo condition and the pair condition. Here, the participants' performance is averaged across 6 sessions. See "numeric simulation about the effect of imitation bias on performance" in the Supplementary Results section for details.

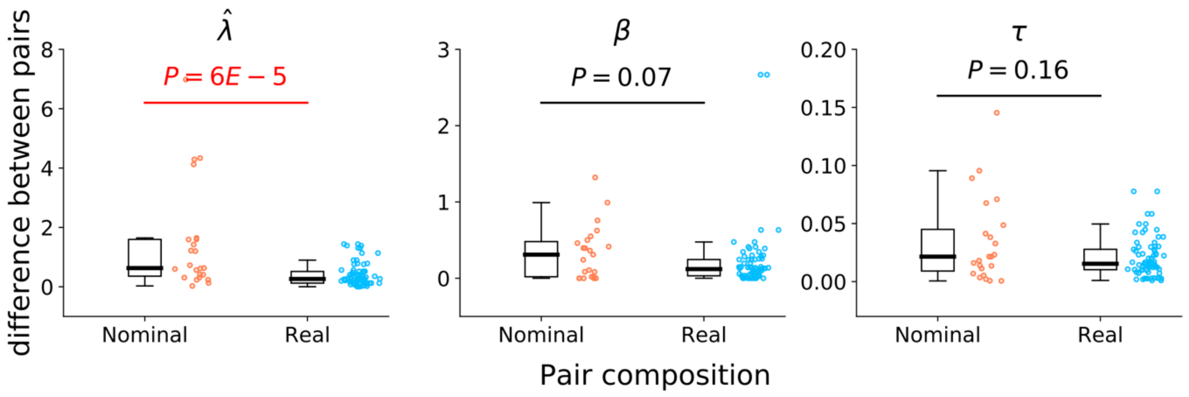

Fig. S8 | Parameter difference between pairs. Nominal pairs (red) consisted of two random participants in the solo condition, while real pairs consisted of those who were actually matched in the pair condition.

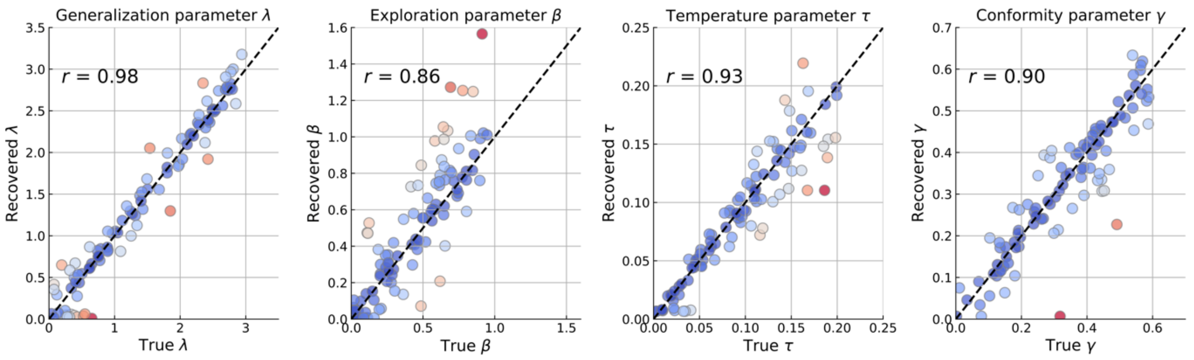

Fig. S9 | Parameter recovery. X-axes show the true parameter values and y-axes the recovered values. The differences between the true and recovered values are colored according to magnitude. The diagonal broken line indicates a difference of zero (i.e., parameters are completely recovered).

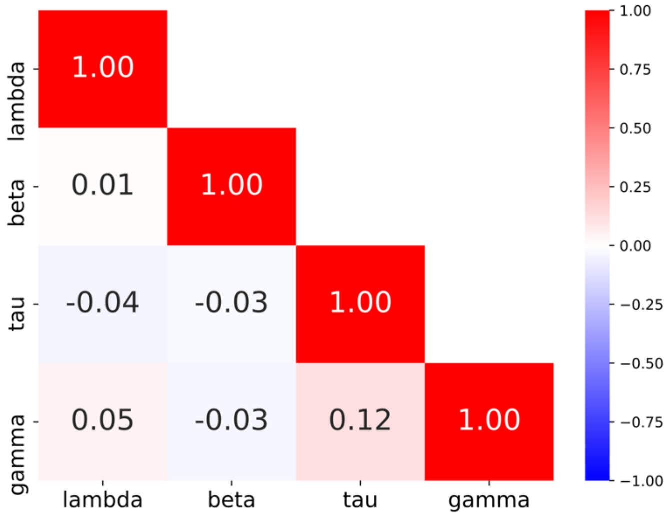

Fig. S10 | Correlations between the recovered parameters.

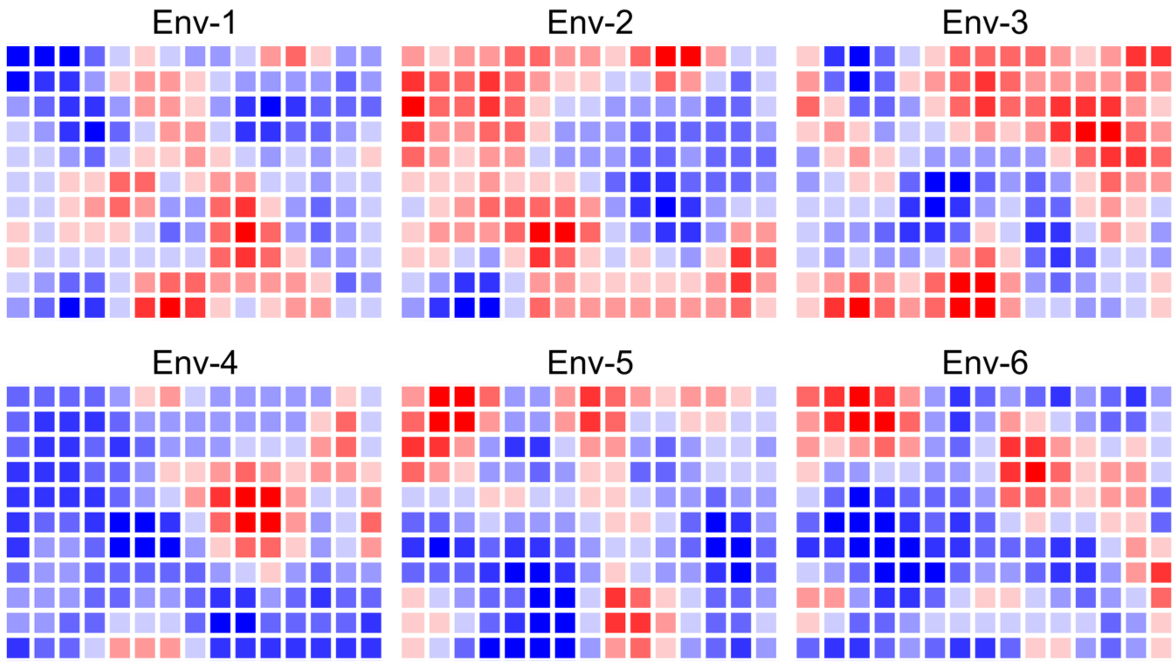

Fig. S11 | High and low reward areas in the 6 environments. Red boxes show options with higher rewards (equal to or higher than 0.5 in the min-max normalization scale) and blue boxes show options with lower rewards (lower than 0.5 in the min-max normalization scale), with darker tones indicating more extreme reward values.

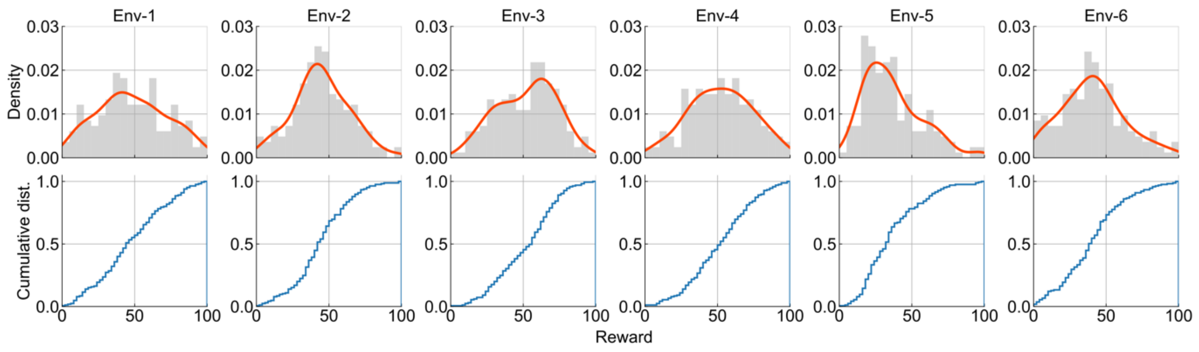

Fig. S12 | Reward distributions in the six environments used in the six main sessions: Histograms and Kernel Density Estimation (KDE) plots (top) and cumulative distributions (bottom).

Supplementary Tables

Table S1 | Mean and 95% Bayesian credible intervals of the estimated coefficients in the LMM predicting the reward earned in each trial in the main sessions.

Effect	Mean	2.5%	97.5%	Effective sample size	$\hat{R}$
β_1 (intercept)	70.28	68.89	71.73	774	1.01
β_2 (session)	-0.02	-0.68	0.63	1286	1.00
β_3 (condition)	3.29	1.45	5.1	853	1.01
β_4 (trial)	1.72	1.55	1.88	1111	1.00
β_5 (session*condition)	1.21	0.31	2.06	1476	1.00
β_6 (condition*trial)	0.26	0.04	0.47	1104	1.00
β_7 (trial*session)	0.06	0.02	0.1	2341	1.00

Table S2 | Mean AICs and model elements

model name	mean AIC	$m(\mathbf{x})$	$\beta \cdot var(\mathbf{x})$	$\gamma \cdot k_{RBF}(x, x_{partner})$
**UCB+S**	966.89	○	○	○
Pure-Exploit+S	969.51	○	−	○
Pure-Explore+S	1295.08	−	○	○
UCB	1031.30	○	○	−
Pure-Exploit	1027.67	○	−	−
Pure-Explore	1515.13	−	○	−
Random	1531.78	−	−	−

Table S3 | Mean and 95% Bayesian credible intervals of the estimated coefficients in the LMM predicting the deviation of subjective predictions about 16 unchosen options.

Effect	Mean	2.5%	97.5%	Effective sample size	$\hat{R}$
β_1 (intercept)	19.06	18.22	19.92	2055	1.00
β_2 (type)	5.97	3.99	7.90	1588	1.00
β_3 (condition)	-0.76	-1.86	0.26	2180	1.00
β_4 (session)	-0.15	-0.46	0.16	3493	1.00
β_5 (type*condition)	-2.03	-4.52	0.46	1751	1.00
β_6 (condition*session)	0.01	-0.38	0.41	3423	1.00
β_7 (session*type)	-0.01	-0.38	0.41	3423	1.00

References

1. Peirce, J. W. PsychoPy--psychophysics software in Python. *J. Neurosci. Methods* **162**, 8–13 (2007).

2. Virtanen, P. *et al.* SciPy 1.0: fundamental algorithms for scientific computing in Python. *Nat. Methods* **17**, 261–272 (2020).

3. Carpenter, B. *et al.* Stan: A probabilistic programming language. *J. Stat. Softw.* **76**, (2017).

4. Wu, C. M., Schulz, E., Speekenbrink, M., Nelson, J. D. & Meder, B. Generalization guides human exploration in vast decision spaces. *Nat Hum Behav* **2**, 915–924 (2018).
